# A Cost-Effective Reference-Less Semiconductor Ion Sensor with Anodic Aluminum Oxide Film

**DOI:** 10.3390/s25216690

**Published:** 2025-11-01

**Authors:** Yiming Zhong, Peng Sun, Zhidong Hou, Mingyang Yu, Dongping Wu

**Affiliations:** 1 State Key Laboratory of Integrated Chips and Systems, College of Integrated Circuits and Micro-Nano Electronics Innovation, Fudan University, Shanghai 200433, China; 20112020045@fudan.edu.cn (Y.Z.); zdhou21@m.fudan.edu.cn (Z.H.); 22212020184@m.fudan.edu.cn (M.Y.); 2 School of Information Technology, Luoyang Normal University, Luoyang 471934, China; sunpeng1@lynu.edu.cn

**Keywords:** pH sensor, anodic aluminum oxide, reference-less

## Abstract

**Highlights:**

**What are the main findings?**

- Developed a low-cost reference-less ion sensor using AAO films.
- Achieved 57.8 mV/pH sensitivity and demonstrated non-destructive monitoring for milk freshness.

**What are the implications of the main findings?**

- Enables miniaturized, cost-effective ion sensors.
- Promises portable, disposable platforms for food safety and diagnostics.

**Abstract:**

The detection and monitoring of ions are essential for a broad range of applications, including industrial process control and biomedical diagnostics. Traditional ion-sensitive field-effect transistors require bulky and expensive reference electrodes, which face several limitations, including device miniaturization, high fabrication costs, and incompatibility with semiconductor manufacturing processes. Here, we introduce a reference-less semiconductor ion sensor (RELESIS) that utilizes anodic aluminum oxide film as both the sensitive and dielectric layer. The RELESIS is composed of a metal-oxide-semiconductor field-effect transistor and an interdigital electrode, which fundamentally eliminates the need for a reference electrode, thereby enabling device miniaturization. During fabrication, the anodic oxidation process is employed in place of the expensive atomic layer deposition method, significantly reducing manufacturing costs while maintaining high surface quality. In practical measurements, the RELESIS device demonstrated an excellent pH sensitivity of 57.8 mV/pH with a low hysteresis of 7 mV. As a proof-of-concept application, the RELESIS device was employed for real-time, non-destructive monitoring of milk freshness, accurately detecting pH changes from fresh to spoiled in milk samples. The combination of reference-less structure, low-cost fabrication, and superior sensing performance positions this technology as a promising platform for next-generation portable ion sensing systems in food safety, environmental monitoring, and point-of-care diagnostics.

## 1. Introduction

Ion monitoring plays a crucial role in modern society, spanning various applications, including industrial process control, environment monitoring, and medical analyses [1,2]. Among various ion sensors, the Ion-Sensitive Field-Effect Transistor (ISFET), first demonstrated by Piet Bergveld in 1970, has garnered significant attention due to its high sensitivity, label-free process, and fast response time [3,4]. The structure of an ISFET is similar to that of a Field Effect Transistor (FET), where the metal gate is substituted with a sensitive electrode in direct contact with the analyte. When ions interact with the sensitive electrode, the surface potential of the solid–liquid interface changes. Furthermore, the surface potential modulates the channel conductance and drain current through the electrical field effect, enabling the detection of target ion concentrations based on variations in the electrical signal [5]. ISFETs have been successfully employed for the detection of a wide range of analytes, including hydrogen ions (H^+^), sodium ions (Na^+^), calcium ions (Ca^2+^), penicillin, and antibodies [6,7,8,9]. In recent years, advancements in functional materials and semiconductor fabrication technologies have significantly improved the sensitivity and integration capabilities of ISFET-based sensors. Among the various applications, pH sensing remains the most mature and widely adopted: in beverages and food, a decreasing pH often indicates declining freshness; in human physiology, the pH of body fluid reflects metabolism, organ function, and disease markers; in industrial processes, pH is a critical control parameter, particularly in fermentation [10,11,12]. However, a stable reference electrode is indispensable for conventional ISFETs to provide a fixed and reliable solution potential during measurements [13]. The widely adopted silver/silver chloride (Ag/AgCl) standard reference electrode offers good stability but suffers from inherent limitations, such as fragility, a lack of miniaturization potential, and difficulty in on-chip integration [14,15,16]. Their relatively high cost—typically several tens of US dollars per unit—also constrains the affordability of ion-sensing systems. Recognizing these limitations, researchers have explored alternatives in terms of electrode materials, structural designs, and operational mechanisms, leading to the development of solid-state reference electrodes, planar reference electrodes, and pseudo-reference electrodes [17,18,19,20,21,22]. Nevertheless, none of the existing alternatives simultaneously addresses all the critical requirements: miniaturization capability, cost-effectiveness, and semiconductor process compatibility.

Under these considerations, our group developed a fundamentally different architecture: the reference-less semiconductor ion sensor (RELESIS), which is inherently distinct from pseudo-reference or REFET approaches [23,24]. In RELESIS, the measurement relies on the potential difference between two non-faradaic, dielectrically coupled interfaces, rather than an absolute solution potential. Consequently, the bulk solution potential is allowed to float freely, and no component in the system functions as a reference electrode. Our initial RELESIS design, reported in 2018, combined an FET with a pair of interdigital electrodes (IDE) [25]. These electrodes are coated with aluminum oxide (Al_2_O_3_) and gold (Au) film, respectively, which exhibit significantly different sensitivities to H^+^. By measuring the potential difference between the IDE rather than measuring absolute potentials, the system eliminates the need for a stable reference potential. While the initial RELESIS device showed promising results, it relied on atomic layer deposition (ALD) for fabricating high-quality Al_2_O_3_ films. Although ALD produces exceptional film quality with precise thickness control and excellent uniformity, this technique requires expensive equipment ($500 K–2 M per system), operates at slow growth rates (typically 0.1–1 nm/cycle), and involves complex precursor chemistry [26]. To further reduce manufacturing costs and improve the scalability of the technology, an anodic oxidation process was employed. This method requires only simple equipment, operates at room temperature, and achieves rapid film growth rates. Previous studies have demonstrated the potential of anodic aluminum oxide (AAO) films in various applications. Chen et al. have reported a humidity sensor based on AAO with good sensitivity and linearity, although the highly porous morphology limits its applicability as gate dielectrics [27]. Cai et al. successfully employed solution-processed Al_x_O_y_ film as the gate dielectric in indium-gallium-zinc-oxide thin-film transistors, achieving excellent electrical performance despite their films being extremely thin (~3 nm) and the composition not being pure Al_2_O_3_ [28]. So far, no previous work has systematically optimized anodic oxidation processes specifically for ion sensing applications, where the films simultaneously serve as both pH-sensitive layers and high-quality gate dielectrics.

In this work, we fabricated a cost-effective, reference-less semiconductor ion sensor employing the optimized AAO film. The proposed RELESIS architecture completely eliminates the need for a reference electrode, enabling miniaturization and compatibility with semiconductor fabrication processes. Furthermore, the device demonstrates near-Nernstian sensitivity (57.8 mV/pH) with low hysteresis (7 mV) and a total fabrication cost below $5 per unit. As a proof-of-concept demonstration, RELESIS was applied to real-time, non-destructive milk freshness monitoring, accurately tracking pH decline during spoilage. The combination of reference-free operation, low-cost fabrication, and strong sensing performance establishes this work as a promising platform for next-generation portable ion-sensing systems in food safety, environmental monitoring, and point-of-care diagnostics.

## 2. Materials and Methods

### 2.1. Sensor Structure and Materials

**Figure 1:**
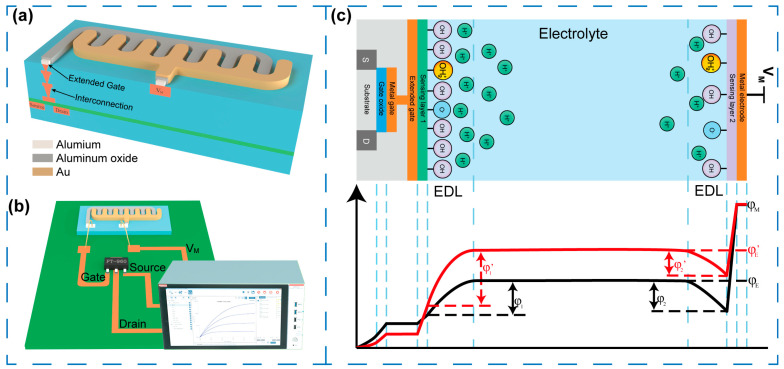
(**a**) Schematic diagram of an integrated RELESIS. (**b**) Schematic of the discrete RELESIS structure and measurement equipment. (**c**) The working principle and potential distribution diagram of RELESIS.

Figure 1a shows the schematic diagram of a completed RELESIS that can be manufactured using mature semiconductor technology. It consists of a metal-oxide-semiconductor field-effect transistor (MOSFET) and a pair of interdigital electrodes. The IDE structure comprises an Al film as the conductive layer and an Al_2_O_3_ film as the dielectric layer. Specifically, the upper comb of the IDE is connected to the gate of MOSFET, while the lower comb is deposited with an additional Au layer. The Al_2_O_3_ film serves as a dielectric layer, ensuring a non-Faradic character and maintaining the stability of the electrodes. In addition, the distinct hydrogen ion sensitivities of Au and Al_2_O_3_ ensure an accurate response of RELESIS. To simplify the fabrication process, a discrete commercial MOSFET (FT960T1, ON Semiconductor Co., Ltd., Phoenix, AZ, USA) was integrated with the IDE to verify the performance of RELESIS. As depicted in Figure 1b, a semiconductor parameter analyzer (4200SCS, Keithley Co., Ltd., Solon, OH, USA) is connected to the MOSFET and IDE through a custom-designed printed circuit board (PCB) during actual experiment measurements. This configuration ensures precise electrical signal acquisition while minimizing parasitic effects during measurement.

### 2.2. Working Principle of RELESIS

The fundamental working principle of RELESIS is schematically illustrated in Figure 1c and described as follows. Unlike traditional sensors that rely on a reference electrode to maintain a stable electrolyte potential, RELESIS functions by measuring the potential difference between two sensitive layers. Sensitive layer 2, which is coated with metallic materials, is insensitive to hydrogen ions, while sensitive layer 1 is sensitive to them. These two sensitive layers are placed close enough so that the applied potential, V_M_, can effectively control the operation of FET. Moreover, the non-faradaic process is a prerequisite of RELESIS; both sensitive layers contain a dielectric layer, ensuring that no current flows across the sensitive layer surface. According to the electrical double layer (EDL) and site-binding model (SBM) theories, when the IDEs coated with sensitive layer 1 (e.g., Al_2_O_3_) and sensitive layer 2 (e.g., Au) are exposed to an electrolyte, surface hydroxylation occurs at the oxide–solution interface, leading to the formation of hydroxyl groups (–OH) that can protonate or deprotonate depending on the hydrogen ion concentration. The surface charge density is governed by the equilibrium of metal oxide groups on the Al_2_O_3_ surface, determining the corresponding surface potential. As pH increases, the deprotonation reaction dominates, resulting in a more negative surface potential. Conversely, at low pH values, protonation occurs, rendering the surface potential more positive. In contrast, the Au surface exhibits weak hydroxyl adsorption and negligible site-binding behavior. Its potential is primarily determined by the electrostatic potential drop across the EDL, with only minimal dependence on the solution pH. Accordingly, the potential distribution profile is depicted in Figure 1c, where the black and red lines represent conditions before and after a pH increase [29,30,31]. In this picture, $\varphi_{s}{,\varphi }_{e},\varphi_{g}$ are the potentials in the semiconductor surface, electrolyte, and extended gate, respectively. Additionally, $\varphi_{1}$ and $\varphi_{2}$ are the potential drop at the electrolyte-sensitive layer interface. Similarly to the theoretical description of an ISFET, a change in pH in electrolyte causes a variation of $\varphi_{1}$ and $\varphi_{2}$, thus, the variation value can be defined as ${\Delta \varphi }_{1}=\varphi_{1}^{\prime }-\varphi_{1}={-2.3\alpha }_{1}\frac{kT}{q}\Delta pH$ and ${\Delta \varphi }_{2}=\varphi_{2}^{\prime }-\varphi_{2}={-2.3\alpha }_{2}\frac{kT}{q}\Delta pH$ respectively. Where *k* is the Boltzmann constant, and $\alpha_{1}(\alpha_{2})$ is the sensitivity factor related to the sensitive layer material. In a giving input potential $V_{M}$, two electrolyte/sensitive layer interface potential (${\Delta \varphi }_{1}\;\mathrm{and}\;{\Delta \varphi }_{2}$) is influenced by the change in pH, thus the system potential distribution is reshaped accordingly. The bulk potential $\varphi_{e}$ and $\varphi_{e}^{\prime }$ is modified through electrostatic equilibrium and capacitive coupling. Consequently, only the interfacial potentials at the sensitive layer/electrolyte interface are directly responsive to variations in pH. Therefore, the change in electrolyte pH can affect the difference in interfacial potential and modulate the channel conductance of FET. The pH sensitivity in the linear regime can be expressed as:

(1) $$\frac{\Delta \varphi }{\Delta pH}=\;\frac{\Delta \varphi_{2}}{\Delta pH}-\frac{\Delta \varphi_{1}}{\Delta pH}=\;-2.3\frac{kT}{q}(\alpha_{2}-\alpha_{1})$$

In Equation (1), the difference in sensitivity factors ($\alpha_{1}\mathrm{and}\;\alpha_{2}$) between the two sensitive layers is a key determinant of sensor performance. The optimal configuration is one in which a sensitive layer is entirely insensitive to hydrogen ions while the other exhibits an ideal Nernstian response. It is worth noting that environmental conditions can influence this mechanism. For instance, temperature variations affect both the Nernstian slope and the dissociation constant of surface hydroxyl groups. Furthermore, prolonged operation in aqueous environments may lead to gradual changes in sensitive surface chemistry or contamination, which can cause slight hysteresis or drift. Overall, under well-controlled experimental conditions, an appropriate combination of sensing-membrane materials (e.g., Al_2_O_3_ and Au) enables a stable, near-Nernstian response, eliminating the necessity for a traditional reference electrode.

### 2.3. Fabrication Process

**Figure 2:**
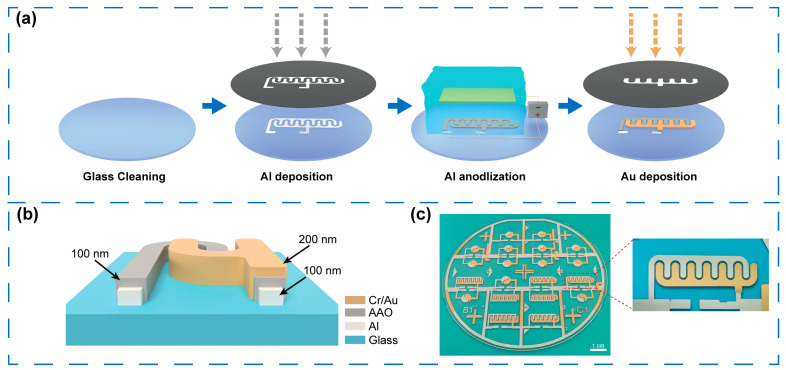
(**a**) Fabrication procedure of IDE, in which two combs were, respectively, coated with two distinct sensitive layers. (**b**) Schematic cross-section of an asymmetric IDE structure with Al, AAO, and Au film. (**c**) Photograph of IDE array fabricated on a glass substrate.

Figure 2a illustrates the detailed fabrication process of the IDE in RELESIS device, using anodic aluminum oxide as both the sensitive layer and dielectric layer. First, a glass wafer with a diameter of 100 mm (Eagle XG, Corning Co., Ltd., NY, USA) was cleaned using a standard cleaning procedure. A 200 nm-thick Al layer was then patterned onto the glass wafer by thermal evaporation using a shadow mask. Subsequently, the Al layer was anodized to form the Al_2_O_3_ gate dielectric. Anodization was performed at 70 V with a current density of 60 mA/cm^2^ in citric acid electrolytes (10 mM/L) at 25 °C under normal atmospheric pressure. Deionized water and ethylene glycol served as the solvent. The Al films were used as the anode, and a graphite plate served as the cathode, with the distance between the two plates kept fixed at 2 cm. In addition, the anodization processes and voltage-time curves were recorded by a programmable DC power supply (PWS4000, Tektronix Co., Ltd., Beaverton, OR, USA). And the experimental setup for the anodizing process is presented in the Supplementary Material as Figure S1. Next, a 5 nm Cr layer, followed by a 200 nm Au layer, was deposited through another shadow mask on one comb of the IDE. The thickness of each film is shown in Figure 2b, and the physical diagram of the IDE is depicted in Figure 2c.

Additionally, the assembly process of RELESIS and test system was illustrated in Figure S2. This step was designed to ensure a stable liquid environment on the surface of the sensitive layer during measurements. Firstly, the glass wafer containing the fabricated IDE structure was diced into smaller substrates (12.5 mm × 22 mm) using a dicing saw (SYJ-400, KJ Group Co., Ltd., Shenyang, China). Then, a dispensing robot (TH-2004D-K, Dahua Co., Ltd., Zhejiang, China) was employed to apply a 0.2 mm-thick epoxy resin around the IDE region, forming a liquid containment chamber that also acted as a buffer sealing layer. After curing the epoxy resin, a custom PMMA cover with microchannels and a PCB testing board were fixed to the glass substrate using screws. Electrical connections between the IDE pads and the PCB circuit were then made with a wire bonder (HS-853A, Baixiangyuan Co., Ltd., Shenzhen, China). Finally, the PCB circuit was connected to the semiconductor parameter analyzer (4200SCS, Keithley Co., Ltd., Solon, OH, USA) through coaxial cables.

### 2.4. Electrical Measurement

The electrical characteristics of the devices were measured using a semiconductor parameter analyzer (4200SCS, Keithley Co., Ltd., Solon, OH, USA). The test electrolyte was commercial buffer solutions (Reagecon, Shannon Co., Ltd., Ireland)) with pH values ranging from 4 to 9. Before pH measurement, all devices were preconditioned by immersion in a pH 7 buffer solution for 1 h to acquire stable EDL interface. In the static measurements for the sensors, the microfluidic system stands by for one minute after each solution exchange. Furthermore, the pH buffer solution is controlled by solenoid valves, while the I-V curves were real time measured.

## 3. Results and Discussion

### 3.1. Morphologies of AAO Film

In a typical anodization process, a bottom barrier alumina oxide and an upper porous alumina oxide are produced successively. Through continued research over the last decades, alumina oxide membranes with different structures can be obtained by controlling experimental conditions (temperature, pH value, anodizing voltage, etc.). Porous AAO films have been widely applied in hard nano-templates and structural coloration due to their high density of ordered pores [32]. In contrast, barrier-type AAO films exhibit excellent insulating properties and high breakdown voltages, making them well-suited for applications in the semiconductor industry [33]. For RELESIS devices, if the membrane is too thin or contains defects, it may allow solution penetration, leading to total device failure. Therefore, in this experiment, a dense and porous-free barrier AAO layer was required as both a sensitive and insulating layer. According to previous experiments, weakly organic acidic electrolytes benefit the production of the barrier AAO film, while ethylene glycol (EG) enables the uniform growth of the alumina oxide [34]. Hence, anodizing was performed in a mixed electrolyte of deionized water and EG with a constant concentration of citric acid (10 mM/L).

**Figure 3:**
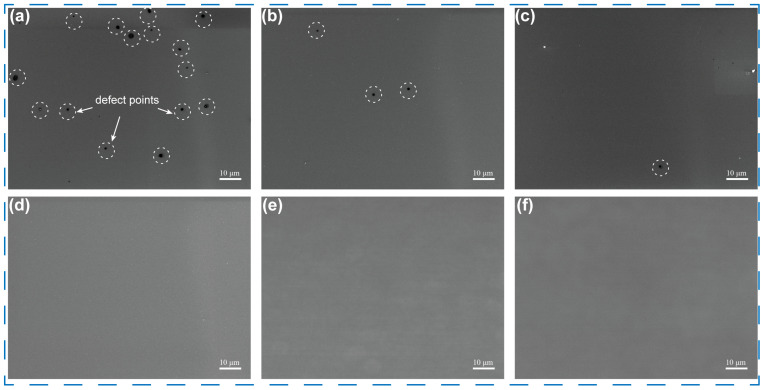
Surface SEM images of the AAO film, which was anodized in citric acid electrolyte with *n*% volume ratio of EG: (**a**) *n* = 0, (**b**) *n* = 10, (**c**) *n* = 20, (**d**) *n* =30, (**e**) *n* = 40, (**f**) *n* = 50.

Figure 3 shows the SEM images of anodic alumina film obtained using citric acid electrolyte with different volume ratios of EG. In Figure 3a, small pores were observed on the surface of the sample using a pure citric acid aqueous solution as the electrolyte. This phenomenon is attributed to internal stress and micro-defects generated during the aluminum deposition process, which leads to non-uniform current distribution during anodization, resulting in over-oxidation and point defects on the film surface. As shown in Figure 3b–d, with increasing EG content in the electrolyte (10–30 vol%), both defect density and size on the film surface decrease significantly. Notably, the point defect disappears entirely when EG content further increases to 30 vol%. This improvement is attributed to EG’s ability to regulate the solubility of aluminum ions, thereby reducing the etching effect of the electrolyte on the oxide layer. When the EG content is further increased to 40–50 vol% (Figure 3e,f), the surface of the anodic aluminum oxide film remains in good condition, with no significant changes. This is because a stable and uniform electric double layer has already formed on the aluminum film surface at a particular EG concentration, facilitating the uniform distribution of free aluminum ions near the anode. Increasing the EG concentration beyond this level does not further enhance the film surface quality. It may even reduce the oxide growth rate due to the decreased mobility of aluminum ions. Figure S3a displays the Atomic Force Microscope (AFM) image of the optimized AAO film, and the surface flatness was evaluated based on the root mean square roughness (*R_q_*). The *R_q_* value of the film surface was measured to be 2 nm, indicating a high degree of smoothness. The Transmission Electron Microscopy (TEM) image shown in Figure S3b also revealed a dense and uniform surface of AAO film. Hence, a 10 mM/L citric acid aqueous electrolyte containing 30 vol% EG was used in the following experiments.

### 3.2. Thickness of AAO Film

**Figure 4:**
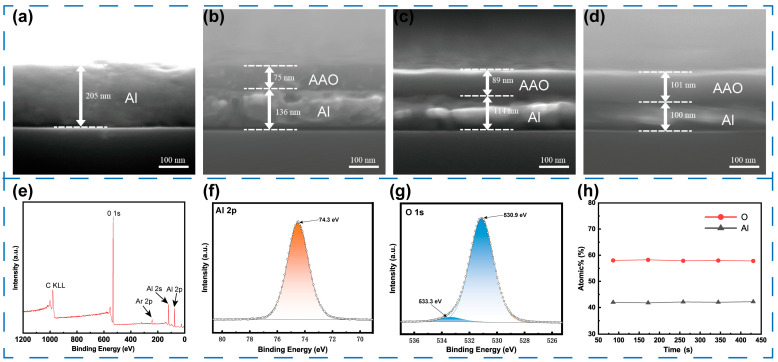
Cross-sectional SEM images of (**a**) Al film and AAO films with the anodization termination current of (**b**) 50 mA, (**c**) 40 mA, and (**d**) 30 mA. XPS spectra of (**e**) survey spectrum (**f**) Al *2p* and (**g**) O *1S* recorded for AAO film. (**h**) The result of atomic concentration profile with depth.

In this RELESIS device, the employed commercial MOSFET gate exhibits an oxide capacitance of approximately 65 pF. To ensure efficient signal transduction, the capacitance of the IDE must be significantly higher than that of the MOSFET gate oxide. When the AAO film thickness is approximately 100 nm, the corresponding IDE capacitance is estimated to be 35 nF, which satisfies the high-capacitance coupling requirement. Moreover, further increasing the oxide thickness leads to a reduction in capacitance due to the inverse proportionality to thickness, ultimately weakening the response. On the other hand, although thinner films (below 50 nm) can improve capacitance, they suffer from reduced dielectric integrity. In such cases, electrolyte penetration and dielectric breakdown were observed after several days during long-term tests, resulting in device failure. Therefore, a thickness of around 100 nm provides an optimal compromise between high capacitive coupling efficiency and long-term stability. For a given anodization potential, the barrier layer thickness (*t_barrier_*) is proportional to the anodization termination current, which corresponds to the total anodization time. Figure 4a–d shows the cross-section SEM images of AAO films prepared with different anodization termination currents. Figure 4b presents the interface of the sample obtained with a termination current of 50 mA, where a clear boundary is observed between the oxide layer and the original Al metal layer. The corresponding barrier-type AAO film exhibits a thickness of 75 nm. When the reaction time is extended and the termination current is reduced to 40 mA, the barrier layer thickness increases to 89 nm. With a further decrease in termination current to 30 mA, the film thickness reaches 101 nm. These results indicate a positive correlation between the reduction in termination current (extended reaction time) and the increase in the thickness of the barrier-type AAO film.

### 3.3. Characterizations of AAO Film

To clarify the surface composition of AAO film, the sample surface was pre-treated by Ar^+^ ion sputtering (2 keV, 10 s) to eliminate surface-adsorbed contaminants and the native oxide layer. Subsequently, X-ray photoelectron spectroscopy (XPS) survey scans were performed to identify the elemental composition of the film (Figure 4e). The spectra revealed that the films were composed mainly of Al and O, indicating high chemical purity. The observed Ar peak results from Ar^+^ sputtering, and the C KLL Auger peak is attributed to minor residual surface carbon, commonly present due to atmospheric exposure. Furthermore, more detailed high-resolution XPS spectra were further acquired for Al and O elements. As shown in Figure 4f, the binding energy of Al-*2p* is 74.3 eV, which is consistent with the Al^3+^ state in typical Al_2_O_3_ (*E_b_* = 74.2–74.6 eV). According to Figure 4g, the O-*1s* spectrum can be decomposed into two peaks: one at 530.9 eV, corresponding to the Al–O bond, and another at 533.3 eV, which is likely attributed to surface-adsorbed hydroxyl groups (H–O bonds) [35]. These features are consistent with the reported characteristics of barrier-type anodic aluminum oxide films. To further investigate whether the elemental composition of the AAO film changes during the growth process, depth profiling analysis was conducted. As shown in Figure S4, the binding energies of Al-*2p* and O-*1s* remain constant throughout the depth of the film. In particular, the Al-*2p* binding energy consistently remains at 74.3 eV, further confirming that the anodic aluminum oxide at different depths primarily exists in the form of Al–O bonds. Figure 4h presents the depth–composition profile of the sample, illustrating the variation in elemental content at different etching times. As observed, the concentrations of Al and O remain stable, indicating that the anodically formed aluminum oxide film is compositionally uniform. And the atomic ratio of Al to O is approximately 2:3, which is consistent with the stoichiometric composition of Al_2_O_3_ [36].

### 3.4. pH Response

**Figure 5:**
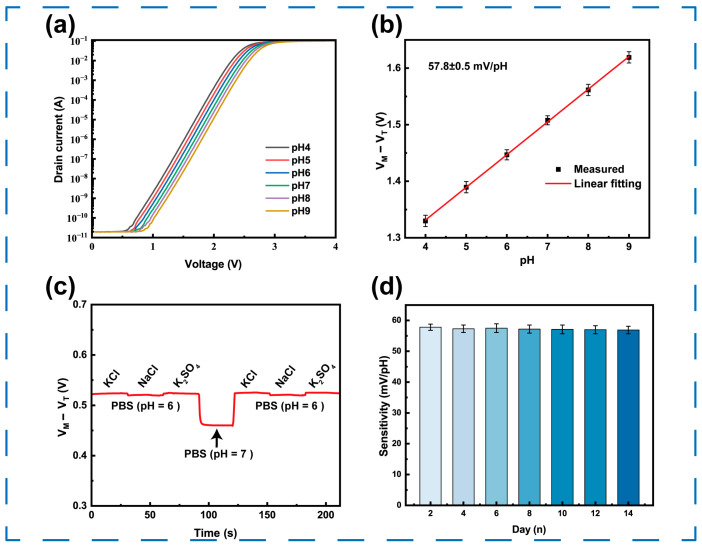
(**a**) Static state transfer characteristics and (**b**) sensitivity characteristics of RELESIS with different pH value buffer solutions. (**c**) Interference effect of K^+^, Na^+^, Cl^−^, and SO4^2−^. (**d**) Long-term stability test.

The pH sensing performance of RELESIS was measured by a microfluidic test system. The drain-source voltage (*V_ds_*) was fixed at 0.1 V, and the modulation voltage (*V_M_*) was swept from 0 V to 4 V in 0.025 V increments. Figure 5a illustrates the transfer characteristics of this device: the transfer curves shift to the right as the pH value increases, indicating that the surface potential of the sensitive layer increases with hydrogen ion concentration. The threshold voltage (*V_t_*) of the RELESIS was defined as the input voltage of the insensitive electrode when the drain current reached 1 × 10^−7^ A. As shown in Figure 5b, the sensitivity was obtained by linearly fitting the *V_t_* with different pH values. And the sensitivity of RELESIS was 57.8 mV/pH for the pH value variation of 4 to 9. This sensitivity closely corresponds to the theoretical sensitivity difference between Al_2_O_3_ and Au film, indicating that the anodically grown Al_2_O_3_ film possesses excellent pH responsiveness and favorable electrical performance.

The hysteresis during dynamic response is another key parameter in evaluating device performance. Due to differences in device structures and measurement conditions, the definition of hysteresis varies across the literature. In this study, hysteresis is defined as the voltage difference between two measurements in neutral buffer solutions (pH = 7), representing the device’s response consistency. As illustrated in Figure S5, the response curve is smooth and stable, indicating good dynamic performance. Moreover, the device exhibits a low hysteresis of 7 mV, which indicates that both the AAO film and the deposited Au layer possess high density and uniformity.

### 3.5. Selectivity and Stability

In addition to sensitivity and hysteresis, the anti-interference capability and long-term stability are crucial parameters for practical applications. During the interference testing process, various interfering ions, including Na^+^, K^+^, Cl^−^, and SO_4_^2−^ were selected for testing. Salt solutions (pH = 6) containing different interfering ions at a concentration of 1 mM/L were prepared. These salt solutions and neutral buffer solution (pH = 7) were sequentially introduced into the microfluidic test system. The results, illustrated in Figure 5c, demonstrate that the output signal shows minimal drift when exposed to these common ions. In contrast, a significant and rapid response was observed only when the pH of the solution was changed. This test confirms that the RELESIS possesses good anti-interference capabilities, ensuring reliable pH measurements in a complex ion environment. Additionally, a 14-day stability test was performed with a standard pH buffer solution, and its sensitivity was measured periodically. As shown in Figure 5d, the sensor maintained excellent stability throughout the testing period, with sensitivity variations less than 2% from the initial value, indicating robust electrical stability of the AAO film.

### 3.6. Comparative Evaluation

To further clarify the novelty and advantages of our device, we added a comprehensive comparison with previously reported FET-based pH sensors. The comparison includes sensing material, fabrication cost, reference electrode requirement, sensitivity, and hysteresis. As summarized in Table 1, conventional ISFET devices typically rely on costly fabrication processes or bulky Ag/AgCl reference electrodes. In contrast, our RELESIS device uniquely combines a reference-less structure with low-cost AAO dielectric/sensitive layers. Notably, this device demonstrates both high sensitivity (close to the theoretical Nernstian limit) and low hysteresis, highlighting its distinct advantage of maintaining outstanding sensing performance while significantly reducing manufacturing costs.

### 3.7. Demonstration

**Figure 6:**
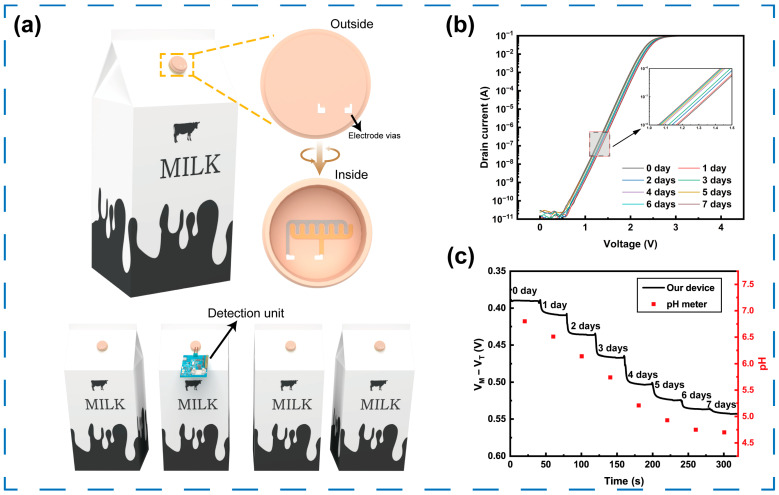
The design concept of RELESIS device in milk freshness detection: (**a**) design schematic of novel carton box which integrates RELESIS sensing area in inner surface, enabling real-time, non-destructive detection of milk freshness. (**b**) Static transfer characteristics and (**c**) dynamic output characteristics of RELESIS with different milk samples, which were stored for n days (n = 0, 1, 2, 3, 4, 5, 6, 7).

Food-quality monitoring has garnered considerable attention in modern society. Milk is widely accepted as a healthy food, but its high nutritional value also contributes to the growth of bacteria that can lead to spoilage [45]. Fresh milk has a nearly neutral pH of 6.5–6.7, but as spoilage progresses, the pH gradually decreases and can even drop to as low as 4 [46]. Therefore, the RELESIS device can be used for real-time measurements of fresh milk. Figure 6a depicts the design schematic of the RELESIS-based milk packaging. The sensing area was fabricated on the inner surface of a milk container lid, while the remaining components of the detection system are provided by a single external detection unit. The sensing module is temporarily connected to the external unit through contact pads on the outer surface of the lid, allowing for the detection of milk freshness without damaging the packaging. Moreover, after measuring one container, the external unit can be quickly repositioned to the next, realizing a single detection unit to identify multiple packaging boxes, significantly reducing operational costs.

As a proof of concept, the RELESIS device was employed to detect milk samples with varying freshness. The milk samples were conditioned in an experimental box with a constant temperature of 25 °C and a relative humidity of 80% to simulate open storage conditions. Milk samples with different storage times (0, 1, 2, 3, 4, 5, 6, and 7 days) were sequentially introduced into a microfluidic system and tested by the RELESIS device. As shown in Figure 6b, the transfer curves shift gradually to the left as storage time increases, indicating a consistent decline in the pH value of the milk. Subsequently, dynamic testing was carried out on the milk samples at different storage times, and at each time point the pH values were also measured using a commercial pH meter for direct comparison. As shown in Figure 6c, the pH value noticeably decreases on the first day. From the second to the fourth day, the rate of decrease accelerates, while from the fifth to the seventh day, the acidic environment inhibits microbial activity, resulting in a gradual slowdown of the pH decline. This trend accurately reflects the expected spoilage process of milk. In addition, the measured data were extracted and normalized, as presented in Figure S6a. The normalized curves clearly indicate that the output of the RELESIS device closely matches the commercial pH meter, with the maximum pH deviation being within ±0.06 pH units, confirming high measurement accuracy. The response time was approximately 8 s (comparable to 3 s for the commercial pH meter), and five repeated measurements in neutral buffer yielded a standard deviation below 2%. Furthermore, after completing the milk tests, four independent RELESIS devices were recalibrated using standard pH buffer solutions to assess their reproducibility. As illustrated in Figure S6b, the sensitivities of the four devices remain highly consistent, indicating superior reproducibility.

## 4. Conclusions

In this study, a cost-effective and high-performance RELESIS device was successfully fabricated using an optimized anodic oxidation process. By incorporating ethylene glycol into a citric acid-based electrolyte, the surface quality of the AAO film was significantly improved, resulting in a dense, pore-free morphology at an ethylene glycol volume ratio of 30%. The film thickness was precisely controlled by changing the terminal anodization current, yielding a uniform 100 nm AAO layer. Therefore, a RELESIS device was fabricated using this optimized AAO film as the sensitive and dielectric layer. In static conditions, the device exhibited excellent pH responsiveness, achieving a near-Nernstian sensitivity of 57.8 mV/pH. In dynamic testing, the device revealed a low hysteresis of 7 mV. Additionally, a practical application was proposed by integrating the RELESIS into a milk carton box, enabling real-time, non-destructive detection of milk freshness. Validation experiments demonstrate that this device could accurately detect pH changes in milk samples at different times, confirming its potential for food quality monitoring. Furthermore, due to its ability to detect pH variations in real time, the RELESIS shows strong potential for deployment in fermentation-based food industries such as yogurt and pickled vegetables.

Despite these promising results, several limitations remain to be addressed. One limitation is that a discrete configuration was employed, in which the IDE and MOSFET were separately assembled to simplify fabrication, rather than being fully integrated on a single semiconductor chip. In addition, although long-term stability was demonstrated under controlled solution conditions, prolonged exposure to complex biological environments may lead to surface fouling or dielectric degradation, potentially affecting sensing reproducibility. Therefore, future work will focus on the semiconductor integration process to realize a fully on-chip RELESIS system. Furthermore, antifouling or self-cleaning strategies will be explored to enhance durability under biofouling-prone conditions, and functionalization of the IDEs will be investigated to enable biochemical sensing. Overall, the combination of low manufacturing cost, reference-less architecture, and extensibility positions this technology as a promising platform for next-generation portable and disposable ion sensing systems in environmental monitoring and point-of-care diagnostics.

## Figures and Tables

**Table 1:** The comparison with other reported FET-based pH sensors.

Sensing Material	Fabrication Method	pH Range	Sensitivity (mV/pH)	Hysteresis (mV)	Cost	Reference Electrode	Ref.
Al_2_O_3_	ALD	3–11	54.7	1.85	High	Ag/AgCl	[37]
AZO	RF Sputtering	1–3	57.9	-	High	Ag/AgCl	[38]
Si_3_N_4_	LPCVD	4–10	58.7	16	High	Ag/AgCl	[39]
APTES/SiO_2_	Thermal oxidation	4–10	50.9	4	Low	Ag/AgCl	[40]
CuS	Solution process	4–10	23.3	0.48	Low	Ag/AgCl	[41]
SiO_2_	LPCVD	4–10	54.8	9	High	Ag/AgCl	[42]
TiN	DC sputtering	2–12	58	2	High	Ag/AgCl	[43]
HfO_2_	ALD	5–9	59.6	44.2	High	Ag/AgCl	[44]
ZnGa_2_O_4_	RF Sputtering	2–12	23.14	-	High	Ag/AgCl	[14]
Ta_2_O_5_	ALD	2–10	56	5	High	Ag/AgCl	[15]
Al_2_O_3_	Anodizing	4–9	57.8	7	Low	Reference-less	This work

## Data Availability

Data are contained within this article.

## Supplementary Materials

The following supporting information can be downloaded at: https://www.mdpi.com/article/10.3390/s25216690/s1, Figure S1: Equipment of anodic oxidation; Figure S2: The assemble process; Figure S3: AFM and TEM image; Figure S4: XPS spectrum; Figure S5: Dynamic measurement; Figure S6: Comparison of RELESIS and commercial pH meter.
