# Supplementary material for: A Cost-Effective Reference-Less Semiconductor Ion Sensor with Anodic Aluminum Oxide Film
Source: Sensors (Basel). 2025 Nov 1;25(21):6690. doi: 10.3390/s25216690 (PMC12609522; doi:10.3390/s25216690)
Supplement: Supplementary file 1 [file sensors-25-06690-s001.zip › sensors-3935010-supplementary.pdf]

Supplementary Material

A Cost-Effective Reference-Less Semiconductor Ion Sensor with Anodic Aluminum Oxide Film

Yiming Zhong [1], Peng Sun [2], Zhidong Hou [1], Mingyang Yu [1] and Dongping Wu [1,*]

[1] State Key Laboratory of Integrated Chips and Systems, College of Integrated Circuits and Micro-Nano Electronics Innovation, Fudan University, Shanghai 200433, China; 20112020045@fudan.edu.cn (Y.Z.); zdhou21@m.fudan.edu.cn (Z.H.); 22212020184@m.fudan.edu.cn (M.Y.)

[2] School of Information Technology, Luoyang Normal University, Luoyang 471934, China; sunpeng1@lynu.edu.cn

* Correspondence: dongpingwu@fudan.edu.cn

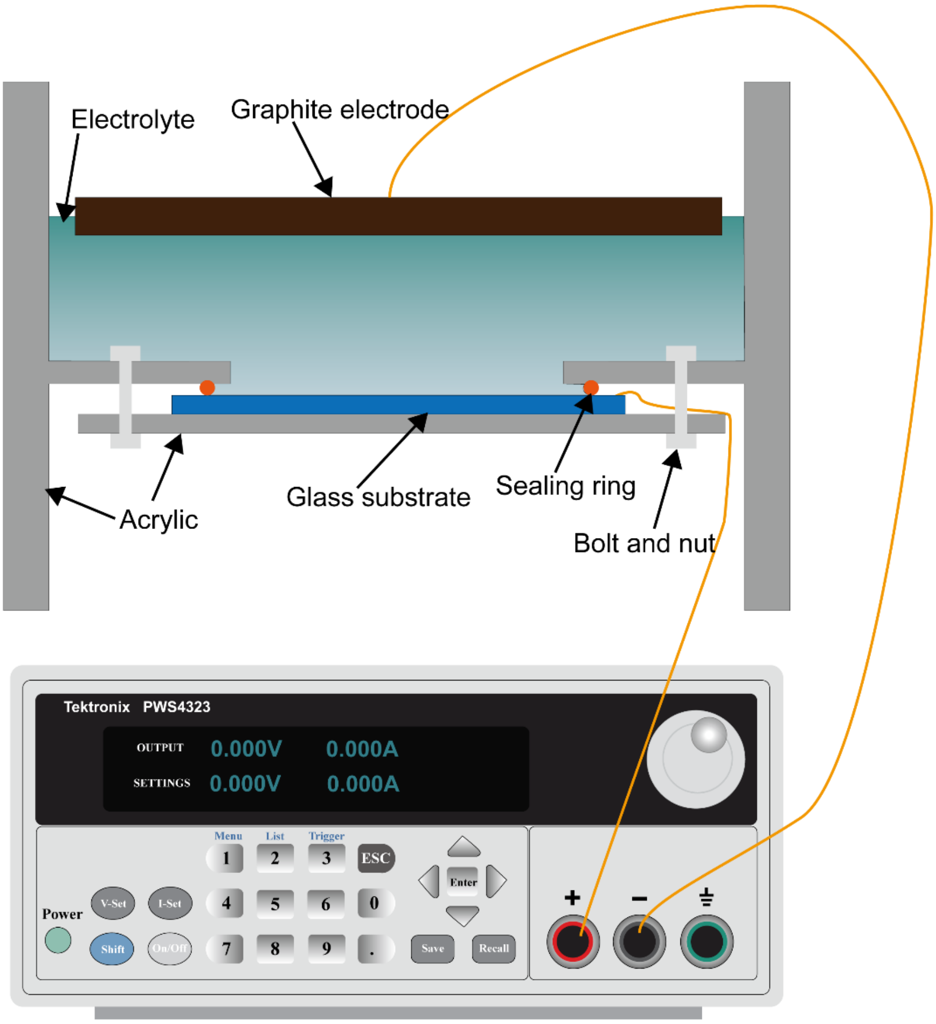

Figure S1. The equipment used in the anodic oxidation process

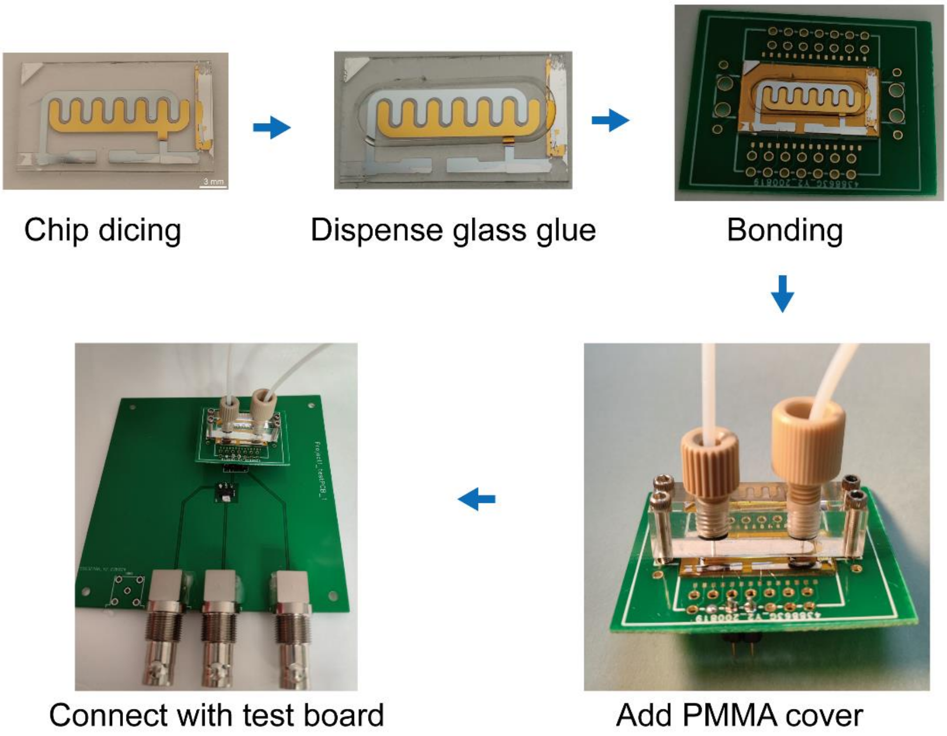

Figure S2. The process of assembling the RELESIS device and integrating it with the microfluidic test system.

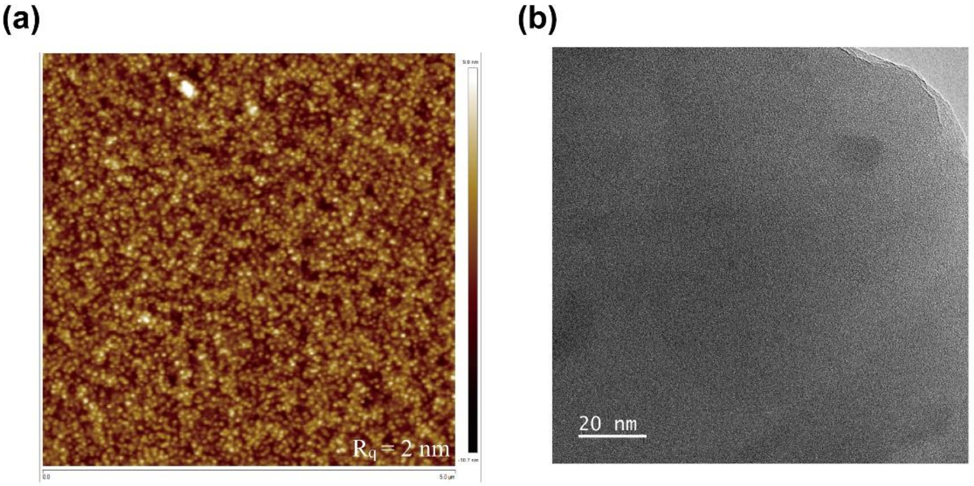

Figure S3. (a) AFM image and (b) TEM image of AAO film

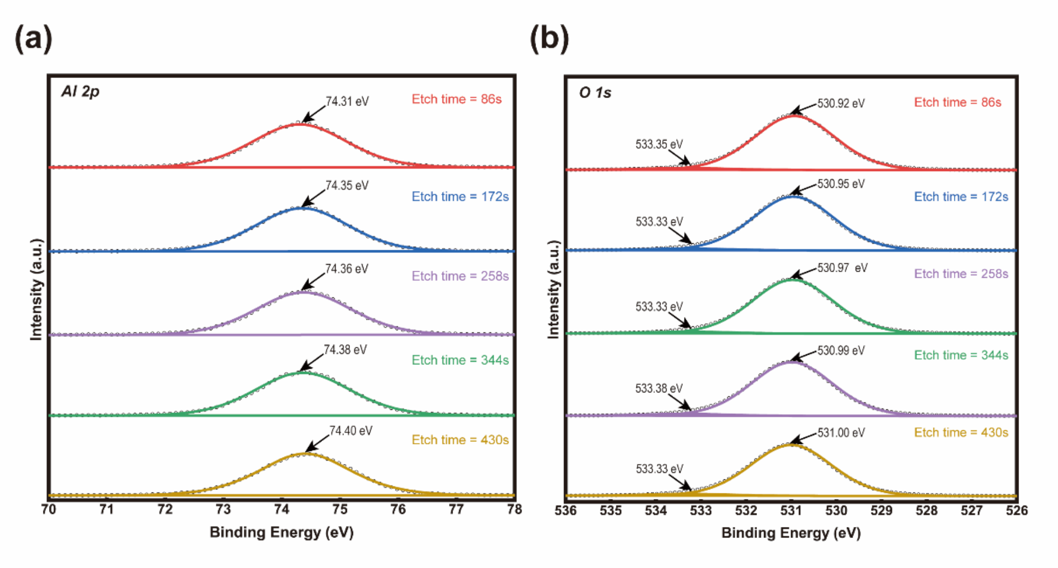

Figure S4. XPS spectrum (a) Al 2p and (b) O 1s of AAO film with different etch time.

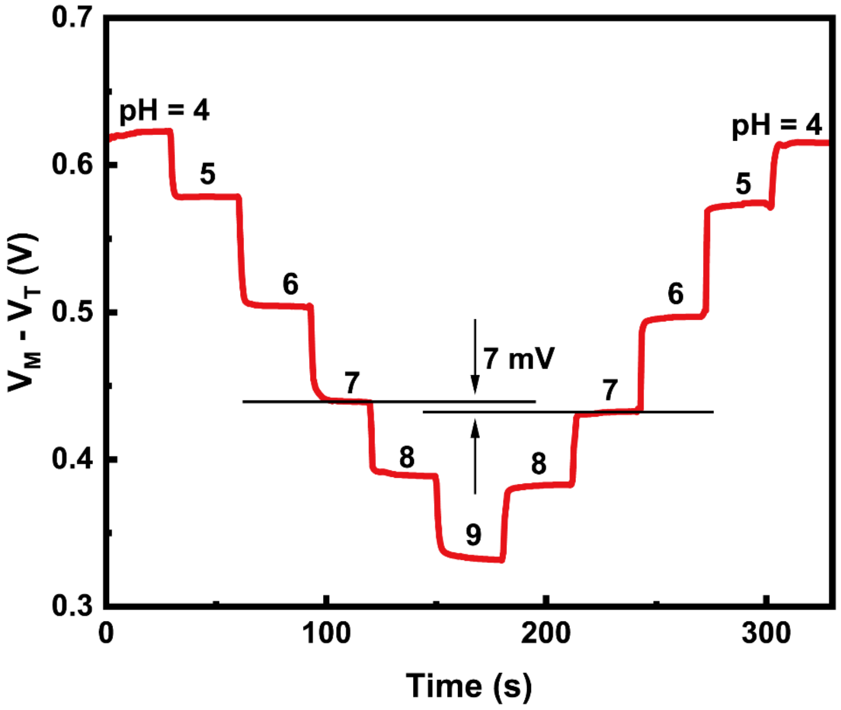

Figure S5. Dynamic measurement result of RELESIS shows low hysteresis

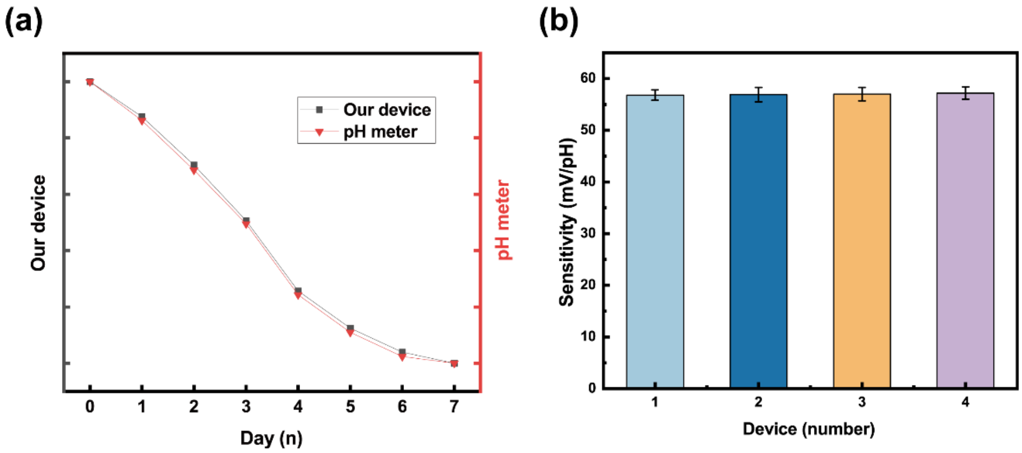

Figure S6. (a) The record result comparison of our device and a commercial pH meter. (b) The corresponding sensitivity of different devices after the milk freshness test.
